# Functional Dynamics of Proteins

**DOI:** 10.1155/2012/242903

**Published:** 2012-12-31

**Authors:** Lee-Wei Yang, Silvina Matysiak, Shang-Te Danny Hsu, Gabriela Mustata Wilson, Yasumasa Joti

**Affiliations:** ^1^Institute of Bioinformatics and Structural Biology, National Tsing Hua University, Hsinchu 300, Taiwan; ^2^Fischell Department of Bioengineering, University of Maryland, College Park, MD 20742, USA; ^3^Institute of Biological Chemistry, Academia Sinica, Taipei 115, Taiwan; ^4^Health Services and Health Administration, University of Southern Indiana, Evansville, IN 47712, USA; ^5^SPring-8/JASRI, Hyogo 679-5198, Japan

X-ray crystallography captures protein conformational states that are stable enough to be captured. These observable states are simply part of the functional event in the context of protein dynamics. Life at the molecular level has a vibrant nature at physiological temperature, governed by physics laws.

Molecular dynamics (MDs) simulations have been used to study biomolecular systems since 1970s [1]. Advancing to this day, it has been a common practice for researchers who access supercomputers to obtain trajectories for hundreds of nanoseconds to two microseconds for small proteins [2, 3] and a few tens of nanoseconds for supramolecular assemblies such as the ribosome of a size larger than 200 Å in explicit solvent with full atomic details [4, 5]. For the few privileged who are able to access machines with build-in hard-wired forcefields, a millisecond trajectory for proteins of ordinary sizes can be made possible [3]. The advancement in parallel computing and hardware speeds enables MD to access timescales of molecular motions that are verifiable by spectroscopy such as IR [6], UVRR [7], FRET [8], and NMR [9]. On the other hand, experimental techniques, especially in X-ray crystallography that has advanced to resolve atomic details for molecules of ever increasing sizes [10], have provided theoreticians a reliable ground to initiate tracking of molecular motions over lengthened time and extended space by leveraging the modern computing environment.

In this issue, we report dynamics stories for biomolecules of different sizes, from crystal waters near the enzyme active sites, “*Implication of crystal water molecules in inhibitor binding at ALR2 active site*,” HIV-inhibiting peptides “*Molecular dynamics simulation of HIV fusion inhibitor T-1249: insights on peptide-lipid interaction,*” V2 vasopressin receptor “*Membrane protein stability analyses by means of protein energy profiles in case of nephrogenic diabetes insipidus,”* ribosomes “*Revealing-1 programmed ribosomal frameshifting mechanisms by single-molecule techniques and computational methods,*” to “walking” myosins “*Coarse-grained simulation of myosin-V movement.*” None of these studies can be made complete with static structures alone. In Hymavati's work, theoretical evidence is presented that proteins bind different ligands (or drug-like molecules) by engaging a variety of water-bridging patterns “*Implication of crystal water molecules in inhibitor binding at ALR2 active site.*” In the MD study led by Luis Loura, potent HIV-inhibitory peptides are found to concentrate near the cell membrane by engaging hydrogen bond contacts with lipids and cholesterol so as to prevent the fusion of HIV envelope with the membrane and therefore increase the antivirial efficacy “*Molecular dynamics simulation of HIV fusion inhibitor T-1249: insights on peptide-lipid interaction.*” Heinke and Labudde demonstrate here how MD can help produce reliable receptor models, whereby stability-related energy profiles can be derived and show how genetic mutations in V2R and aquaporin-2 impair their stability and functions, which leads to the disordered phenotype, nephrogenic diabetes insipidus (NDI) “*Membrane protein stability analyses by means of protein energy profiles in case of nephrogenic diabetes insipidus*”. In similar vein, Zhang et al. offer a comprehensive review on how missense mutations could impact protein stability and dynamics and therefore function “*Analyzing effects of naturally occurring missense mutations.*” 

Despite of the great potential of MD, protein size can easily limit its applicability. It is certainly challenging to simulate macromolecules for timescales of immediate biological interest. The problem can be worsened with inherited sampling problems [11]. The directions of initial momenta of atoms are not specifically assigned (although the magnitude of velocity is deterministic at given temperatures), and therefore a few trajectories have to be obtained to ensure an unbiased sampling. 

Coarse-grained (CG) modeling and simulation techniques emerged in recent years as a promising alternative to obtain biologically relevant dynamics for large macromolecules [12–19]. Dynamic nature and functional details of ribosomes, myosins or even virus capsids of a size as large as 675 Å “*Coarse-grained simulation of myosin-V movement,” “Analyzing effects of naturally occurring missense mutations*” [20] can now be theoretically studied thanks to the development of CG techniques. The computational speed of elastic network models (ENMs), a normal mode analysis for proteins in their CG-ed presentation, can be made five orders of magnitude faster than MD simulations [12–14, 21, 22]. The elastic description of residue-residue interaction reflects the physical nature of folded proteins that sample their surrounding energy landscape [14, 22]. Due to the elimination of fine interaction details, the potential energy surface underlying the conformational transition is smoothened, and the sampling for global dynamics at the expense of atomistic details is enhanced [22, 23]. ENMs have demonstrated that the observed “bound” structures have been readily accessible to the “unbound” structures by ENM-inferred low-frequency normal modes [14, 22, 24], while the “induced fit” can be made apparent to dominate the conformational transitions in strong ligand-protein interactions [25] (Figure 1).

Aforementioned physical nature can be well explored for supramolecular assemblies with a size of the ribosome by atomistic MD simulations and CG-techniques. Chang gives a comprehensive review in this issue on the possible helicase activity in ribosomes and a phenomenon that is called −1 programmed ribosomal frameshifting (−1 PRF), the functional dynamics of which is a realization of “conformational selection” and “induced fit.” The review covers recent progresses on single molecular and modeling techniques, especially the optical tweezers, fluorescence (Förster) resonance energy transfer (FRET), and ENM, followed by the active uses of these techniques to unravel the mystery of −1 PRF, an elaborate and efficient use of one messenger RNA to produce two or more gene products of totally different amino acid sequences and structures so as to maintain life.

Katsimitsoulia and Taylor herein report a study using coarse-grained hierarchical models, at three different structural resolutions, to simulate Myosin's “walking” on the actin filaments. The simulations reproduce observed mean length of a processive run for myosin V, estimated at 2.4 *μ*m, or approximately 66 steps of 36 nm each. The simulations can potentially model more than a hundred of such macromolecules using only 8–12 cpu cores, as the article suggested. The progress in simulation techniques along with fast and parallel computing facilities is gradually bridging the gaps between molecular and cellular simulations. Before another decade, we may expect to see married molecular dynamics and systems biology simulations developed to bring unprecedented mechanistic insights of life at the molecular and cellular levels.

*Lee-Wei Yang* *Silvina Matysiak* *Shang-Te Danny Hsu* *Gabriela Mustata Wilson* *Yasumasa Joti*

## Figures and Tables

**Figure 1:**
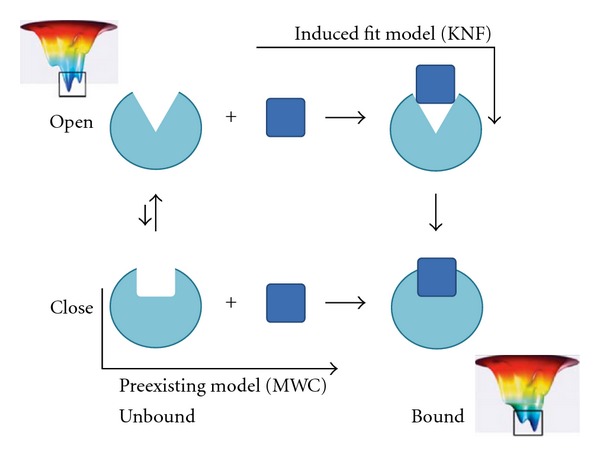
Induced fit versus conformational selection. Protein (in cyan) exists preexisting conformers and “selects” a given conformer by stabilizing it with the incoming ligand (in blue). ENM reveals such transition via its low-frequency normal modes. KNF, Koshland-Néméthy-Filmer; MWC, Monod-Wyman-Changeux.
